# Using 3D-printed fracture networks to obtain porosity, permeability, and tracer response datasets

**DOI:** 10.1016/j.dib.2023.109010

**Published:** 2023-02-25

**Authors:** Megumi Konno, Alexandros Patsoukis Dimou, Anna Suzuki

**Affiliations:** Institute of Fluid Science, Tohoku University, 2 Chome-1-1 Katahira, Aoba-ku, Sendai, Miyagi 980-8577, Japan

**Keywords:** Additive manufacturing, Fractured media, Flow experiment, Fluid Flow, Fluid Transport, Geoscience, Species Transport

## Abstract

An in-depth understanding of flow through fractured media is vital to optimise engineering applications, including geothermal energy production, enhanced oil recovery, CO_2_ storage, and nuclear waste disposal. Advances in 3D-printing technologies have made it possible to generate 3D printed fracture networks with different fracture characteristics. By performing fluid flow experiments in the 3D-printed fractured networks, the impact of the fracture parameters, such as the density, orientation, aperture, dip, and azimuth, on the overall flow can be investigated. This data article contains a detailed description of the framework followed to design fractured networks with different fracture parameters and to create 3D-printed samples, including fracture networks. Furthermore, it contains the experimental protocols used to measure the porosity, permeability, and tracer responses of the 3D-printed samples. The generated datasets provided include geometry data describing the fracture networks, as well as porosity, permeability and tracer response data obtained from flow experiments conducted in the fracture networks.

**Specifications Table**

**Table:** 

Subject	Earth and Planetary sciences, Engineering, Energy

Specific subject area	Fluid flow through fractured media
Type of data	CSV (Fracture information (center, radius, aperture, dip, azimuth)) STL (Fracture network 3D geometries) SCAD (Script for generation of fracture networks) Text (Tracer response) Xlsx (Permeability data, Porosity data) Table Figure The three-dimensional STL files can be viewed using software such as Microsoft 3D viewer, Paraview, MeshLab, 3D Builder or Autodesk Fusion 360. Furthermore, the STL files can be uploaded directly to 3D printing software for 3D printing. The SCAD files can be used for generation of fracture network geometries using the OpenSCAD free software.
How the data were acquired	Generation of 3D fracture networks: Use of OpenSCAD software. Generation of 3D printed fracture network samples: Use of 3D-printer AGILISTA 3100 (KEYENCE CO.). Porosity measurements: Measure the weight of the dry and wet samples with a scale. Equipment used: Desiccator (2-931-04 Forming Vacuum Desiccator (AS ONE Corp.), rotary vane pump (RV Two Stage Rotary Vane Pump (Edwards Corp.), Scale. Permeability measurements: Measure the pressure differential between the inlet and outlet for given flowrates. Equipment Used: rotary vane pump (RV Two Stage Rotary Vane Pump (Edwards Corp.), pressure regulator, 3mm ID tubing. Tracer response measurements: Analyzing the fluid at the outlet of the sample and measuring tracer (Na+) concentration. Equipment Used: Sodium ion (Na^+^) concentration sensor (LAQUAtwin Na-11 (Horiba Co.)), rotary vane pump (RV Two Stage Rotary Vane Pump (Edwards Corp.), 3mm ID tubing.
Data format	Raw Analyzed
Description of data collection	The fracture network data are generated using the OpenSCAD code provided with this work. The porosity data are calculated by measuring the dry and fully water saturated 3D printed samples. The permeability and tracer response data are collected by performing fluid flow experiments in 3D printed samples under room temperature conditions.
Data source location	Institution: Institute of Fluid Science, Tohoku University City/Town/Region: Aoba-ku, Sendai, Miyagi Country: Japan
Data accessibility	Data: Repository name: Open Science Framework Data identification number: DOI:10.17605/OSF.IO/ZN7PA Direct URL to data: https://osf.io/zn7pa/

## Value of the Data

• Experimental data presented in this paper offer benchmark experimental datasets to validate theoretical/numerical models for fluid flow in fractured networks.
• Understanding fluid flow in fractured media and building accurate numerical models is essential to optimise engineering applications, including contaminant transport, geothermal energy, enhanced oil recovery, CO_2_ storage, and nuclear waste disposal.
• Geometry data of fracture networks provided with this dataset can be used to generate 3D printing fracture network samples in order to perform experimental/numerical investigation of further fluid flow processes encountered in fractured media, such as thermal transport, reactive transport and multiphase flow. Furthermore, the code provided allows for altering the fractured networks used in this work.

## 1 Objective

The objective of this work is the development of benchmark experimental datasets which can be used to validate theoretical/numerical models describing fluid flow in fracture networks. 3D printing allows for the generation of three-dimensional complex fracture networks offering geometry control over the fracture network geometrical properties (e.g., fracture density, azimuth, density, aperture, and dip). Furthermore, 3D printing has been validated as a method of generating devices for fluid flow experiments [1]. By performing porosity and permeability measurements, in addition to tracer response experiments using the 3D-printed fracture network models, the impact of the fracture network properties on the overall flow can be investigated. Therefore, benchmark experimental datasets that could be used to validate theoretical/ numerical models can be obtained. The development and validation of the theoretical and numerical models describing fluid flow in fracture networks will allow optimization of engineering applications such as CO_2_ storage, geothermal energy, and contaminant transport.

## 2 Data Description

### 2.1 3D-Printed Fracture Network

**Fig. 1:**
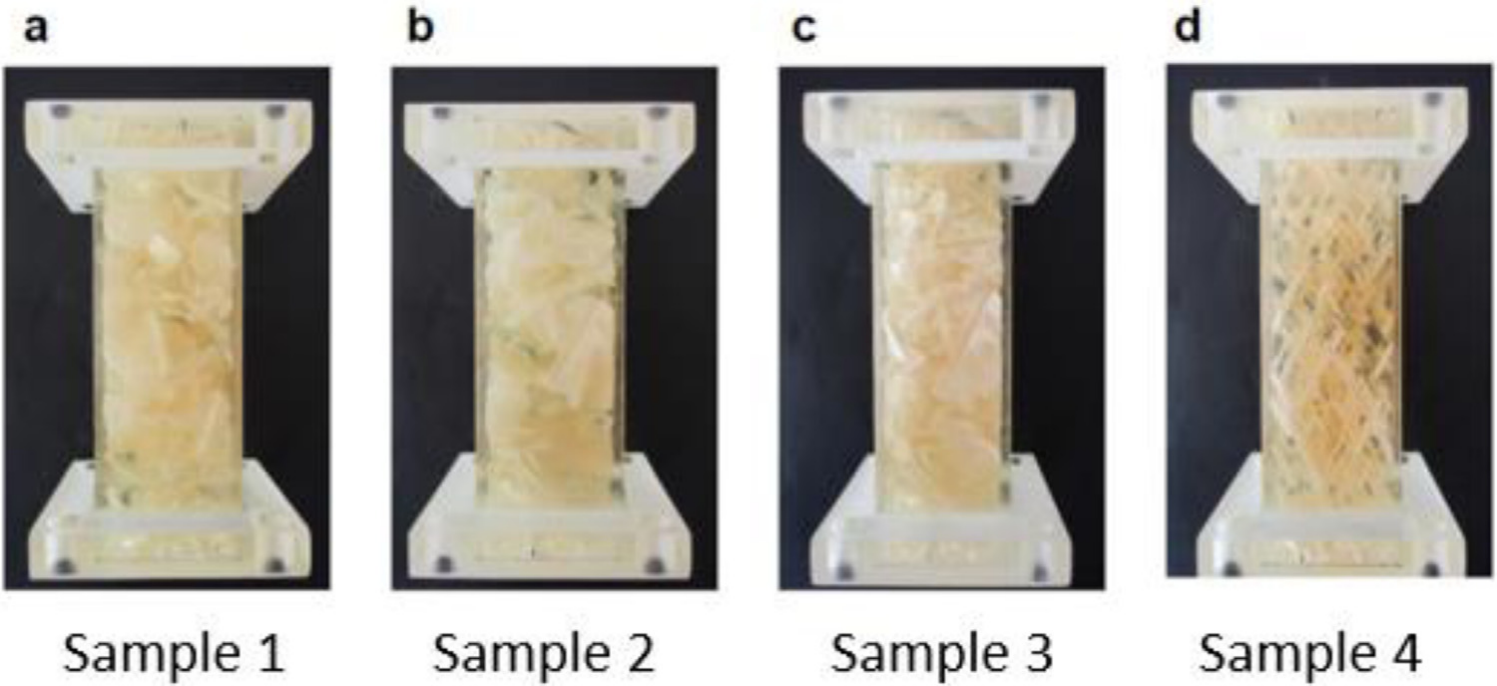
3D-printed fracture network samples generated with 3D-printing.

The dataset contains geometry data of four 3D-printed fracture networks, including: (1) three-dimensional digital geometries of the fracture networks (.stl), (2) datasheets including fracture centres, radius, aperture, dip, and the azimuth of each fracture (.csv). Moreover, it contains (3) OpenScad scripts used for the generation of the geometries. The datasets and OpenSCAD scripts can be found in [2]. The 3D-printed network geometries can be seen in Fig. 1.

### 2.2 Porosity Data

The porosities of the samples are listed in Table 1. The parameters used for calculating porosity, such as density *ρ* (g.m^−3^) and viscosity *µ* (Pa.s) of the water, are listed in Table 2.

**Table 1:** Dry weight, wet weight, and porosity of the 3D-printed samples.

	Sample 1	Sample 2	Sample 3	Sample 4
$w_{dry}$(g)	156.99	163.36	153.55	161.85
$w_{sat}$(g)	176.11	179.19	175.33	178.71
Difference (g)	19.12	15.83	21.78	16.86
Porosity (-)	0.199	0.165	0.227	0.175

**Table 2:** Parameters for the calculations.

Parameter	*µ* (Pa.s)	ρ (g.m^−3^)	*A* (m^2^)	*Δx* (m)
*Value*	8.90 × 10^−4^	10^6^	9.61 × 10^−4^	0.1

### 2.3 Permeability Data

**Fig. 2:**
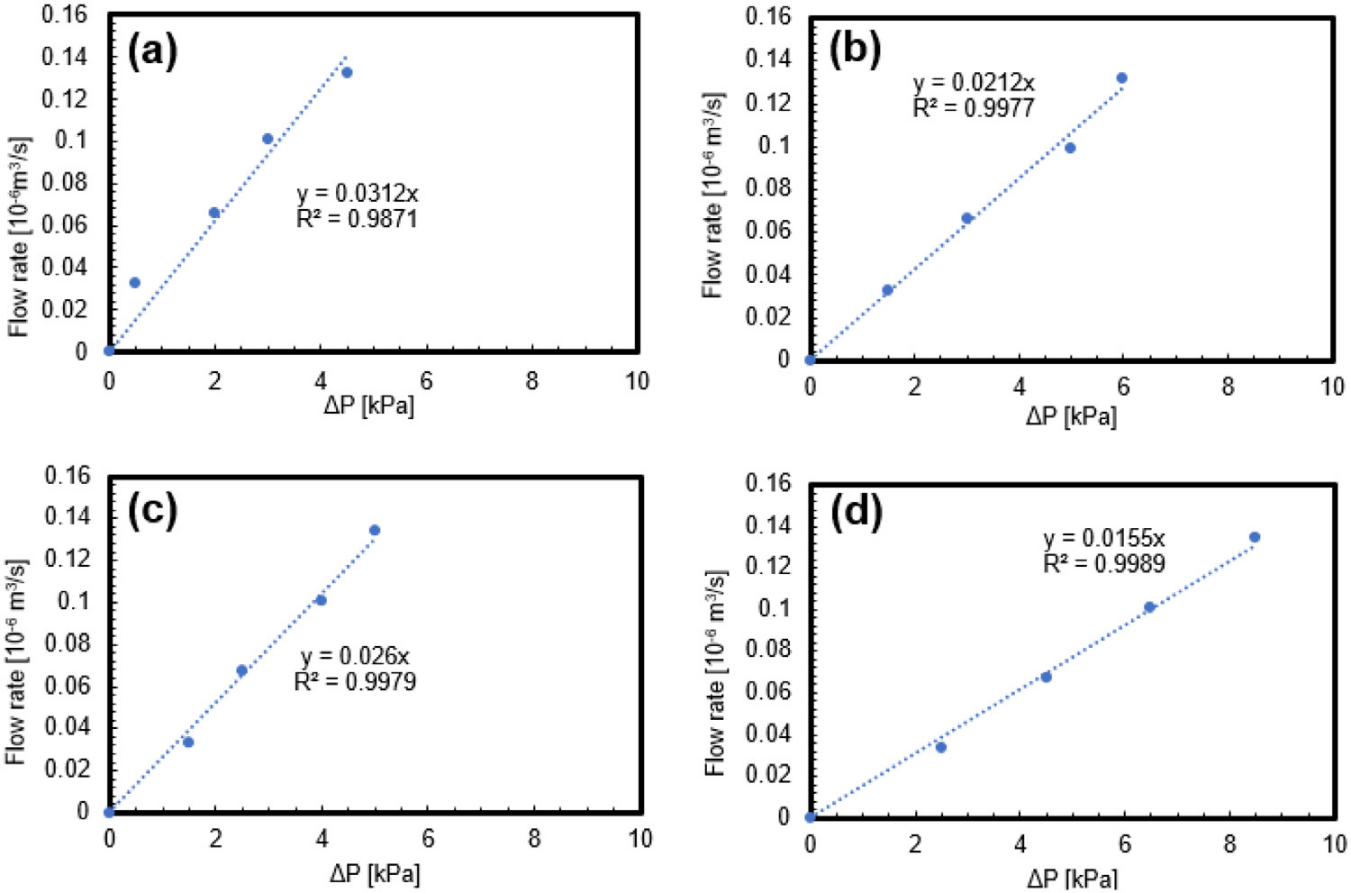
Flow rate vs pressure difference. (a)-(d) Samples 1-4.

The relationship between pressure difference ΔP(kPa) and flowrate *Q* (m^3^.sec^−1^) obtained from the flow experiments using the 3D-printed fracture network samples is presented in Fig. 2. The parameters used for calculating permeability, such as cross-sectional area *A* (m^2^) and length of the samples *Δx* (m) are also listed in Table 2. The measured permeability values for the different 3D-printed fracture network samples are summarised in Table 3. Moreover, the raw data can be found in the data repository (Permeability_data.xlsx).

**Table 3:** Permeability values of the 3D-printed fracture network samples.

Sample	Measured Permeability (m^2^)
1	2.889 × 10^−12^
2	1.963 × 10^−12^
3	2.408 × 10^−12^
4	1.435 × 10^−12^

### 2.4 Tracer Response Data

**Fig. 3:**
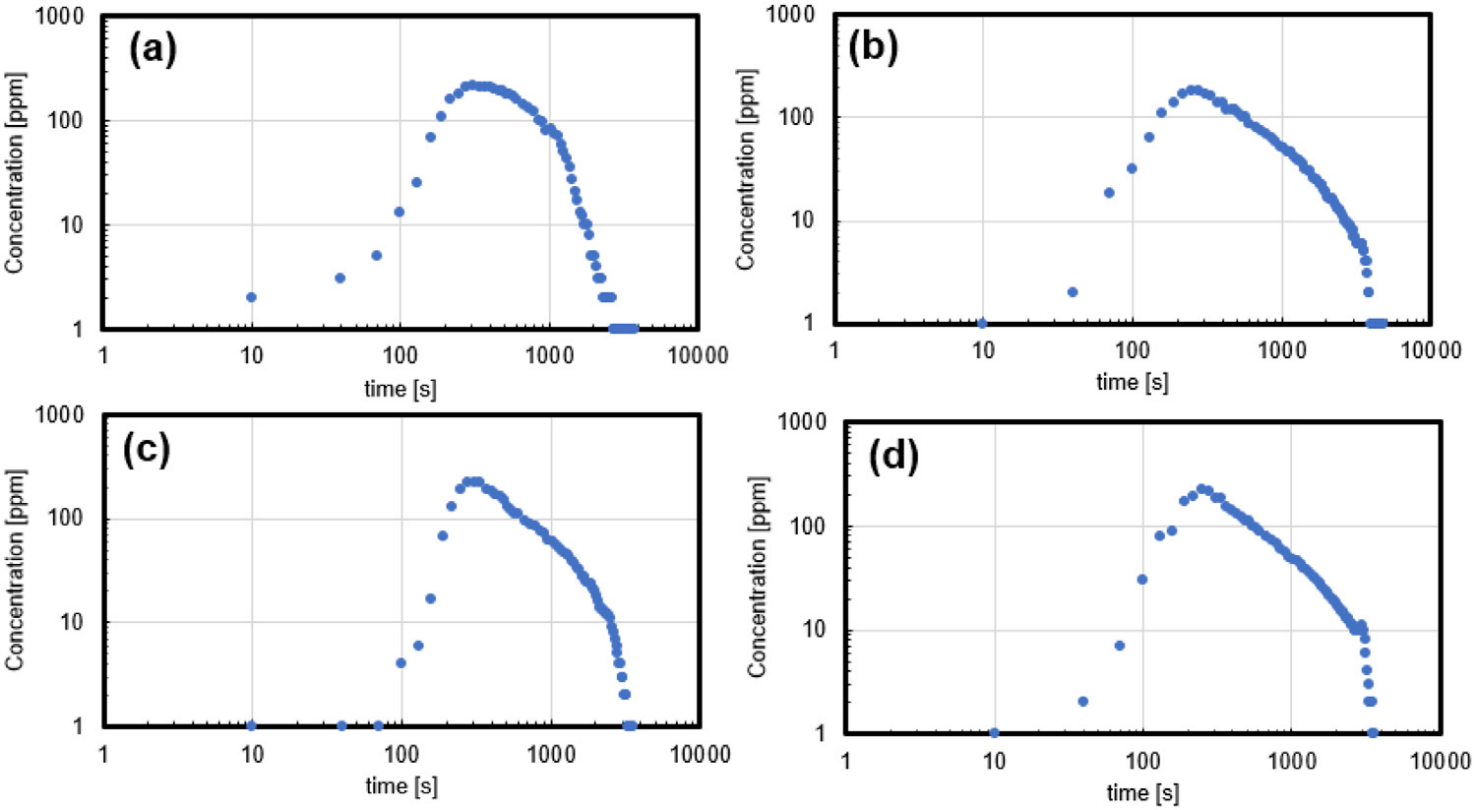
Tracer responses obtained from the 3D-printed fracture network samples: Sample 1-4(a)-(d).

The temporal evolutions of Sodium ion (Na^+^) concentration (ppm) measured at the outlet of each 3D-printed sample, obtained from the tracer response experiments, are shown in Fig. 3. Furthermore, the raw data are provided in the data repository in the tracer response folder for each sample.

## 3 Experimental Design, Materials and Methods

### 3.1 Generation of fracture networks

A detailed description of the fracture network generation can be found in [3]. The fracture networks were formed by disc-shaped fractures. The radii of the disc-shaped fractures were specified according to the power-law scaling with maximum and minimum radii. The fracture aperture was specified to be proportional to its radius based on linear elastic fracture mechanics [4]. The values of maximum and minimum radii were set to 15 mm and 1.6 mm, respectively. The ratio of fracture aperture to the length was set to 0.125, which was determined due to the limitation of the sample sizes and the precision of the 3D-printer. Fractures generated in this study were allowed to cross each other.

Fractures were generated until the fracture density reached the determined level with power-law fracture length distribution. The number of fractures in each sample *N* is given by

(1) $$N=C\left[r_{min}^{-D}-r_{max}^{-D}\right].$$

where *C* is the fracture density parameter, *D* is the fractal dimension, $r_{min}$ (m) and $r_{max}$ (m) are the specified radii of the smallest and largest fracture in the sample, respectively [5,6]. The locations and orientations of the fractures were randomly generated. The fracture network parameters for the four different samples generated can be seen in Table 4.

**Table 4:** Description of the 3D-printed fracture networks.

	Sample 1	Sample 2	Sample 3	Sample 4
C	11050	17700	25000	20000
D	2	2.5	2.5	2.5
Radius	Power law	Power law	Power law	Power law
$r_{max}$	15	15	15	15
$r_{min}$	1.6	1.6	1.6	1.6
A	0.125	0.125	0.125	0.125
Orientation	random	random	random	60^o^

The fracture network geometries were printed using the inkjet 3D-printer AGILISTA 3100 (KEYENCE CO.). The AGILISTA 3100 uses two types of resin materials when performing the print, AR-M2 transparent UV-curable resin for the final geometry and AR-S1 for the support material, which is water soluble and is removed to reveal the final geometry by inserting the print into distilled water after printing [7]. The printer's resolution is 635 × 400 dpi, and the resolution at the *X*-axis is 15 μm.

### 3.2 Porosity Measurements

**Fig. 4:**
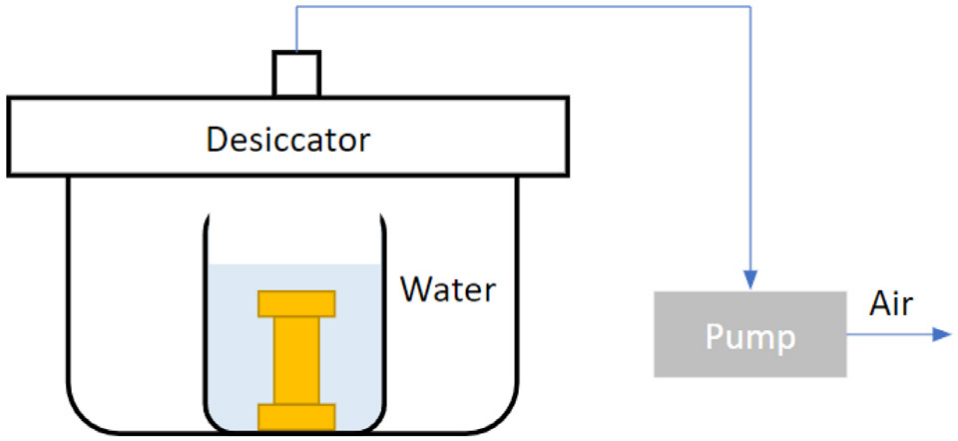
Experimental apparatus for porosity measurement.

The experimental protocol for the porosity measurements of the 3D-printed samples can be seen in Fig. 4. Firstly, the weight of the dried samples (i.e., dry weight) *w_dry_* was measured*.* Each sample was then placed in a beaker and filled with distilled water. The beaker was placed in a Desiccator (2-931-04 Forming Vacuum Desiccator (AS ONE Corp.)), which was connected to a rotary vane pump (RV Two Stage Rotary Vane Pump (Edwards Corp.)) and left to degas overnight. After the degassing process was completed, the sample was removed from the vacuum desiccator, the sides of the sample were wiped, and the weight of the saturated specimen with water (i.e., wet weight) *w_sat_* was measured. The porosity can be calculated as

(2) $$Porosity=\;\frac{\frac{\left(w_{sat}-w_{dry}\right)}{\rho_{water}}}{V},$$

where *V* is the volume (m^3^) of the sample and $\rho_{water}$ (kg.m^−3^) is the water density.

### 3.3 Permeability Measurements

**Fig. 5:**
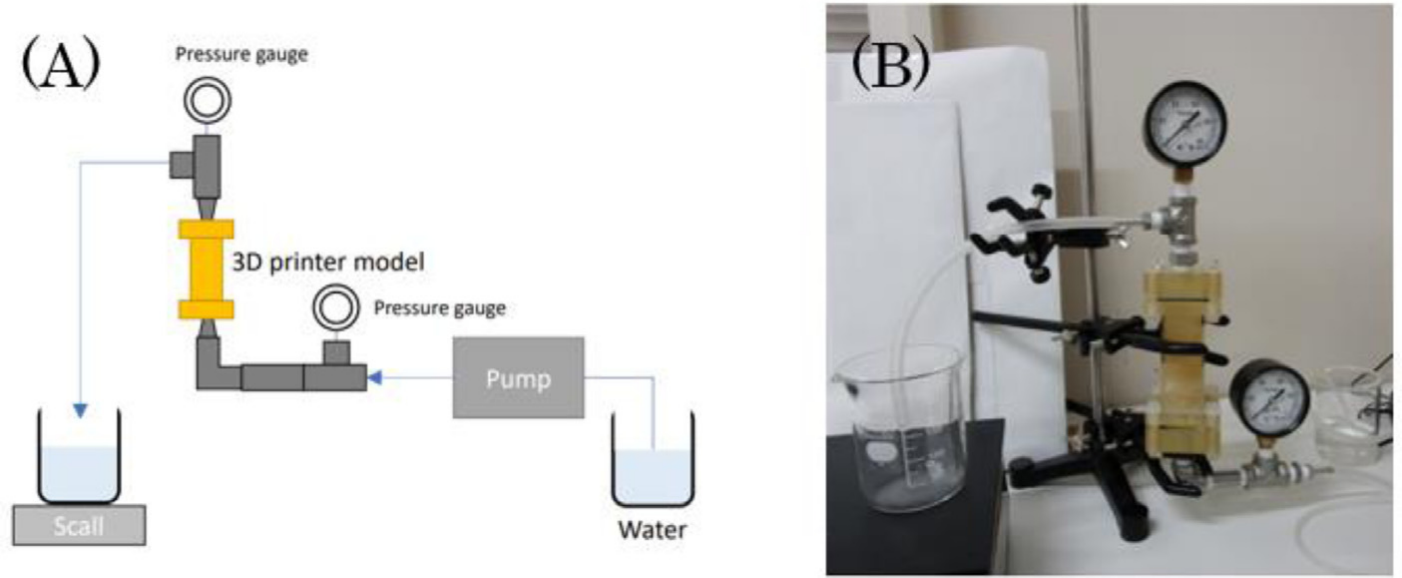
(A) Schematic and (B) image of the experimental apparatus used for permeability measurement of the 3D-printed fracture network samples.

The experimental apparatus for the permeability measurements can be seen in Fig. 5. A container with distilled water was connected via 3mm ID tubing to a rotary vane pump (RV Two Stage Rotary Vane Pump (Edwards Corp.)), to a pressure regulator, and the inlet of the 3D-printed sample. The sample outlet was connected via a 3mm ID tubing to a pressure regulator and a waste container. A flow test was then conducted by injecting distilled water at a constant flow rate *Q* (m^3^.sec^−1^)*.* For each sample, four flow experiments were conducted at four different flow rates, and the pressure difference ΔP between the inlet and the outlet (Pa.s) was measured. Using the parameters from Table 2, the permeability *K* (m^2^) of the sample can then be calculated as [8]

(3) $$K=-\frac{Q*\mu *\Delta x}{A*\Delta P},$$

where ΔP (Pa.s) is the pressure difference between inlet and outlet, μ (Pa.s) is the dynamic viscosity and Δx (m) is the length of the sample and *A* (m^2^) is the cross-sectional area.

**Fig. 6:**
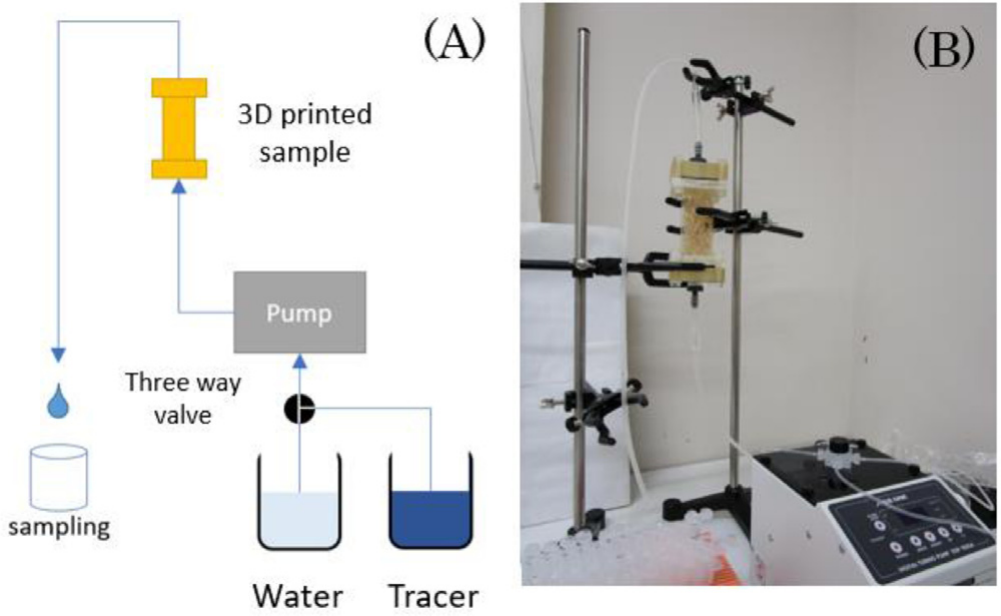
(A) Schematic and (B) photo of the experimental apparatus used for the tracer response measurements with the 3D-printed fracture network samples.

### 3.4 Tracer Response Measurements

The experimental apparatus for the tracer response measurements can be seen in Fig. 6.

A container with distilled water and a container with distilled water seeded with 9000 ppm of Sodium ion (Na^+^) tracer were connected via a 3mm ID tubing and a three-way valve, to a tubing pump (1-5916-01 Digital Quantitative Tubing Pump DSP -100S (AS ONE Corp.)). The pump was connected via a 3 mm ID tubing to the 3D-printed fracture network model. Firstly, the model was saturated with distilled water by injecting with a constant flow rate of *Q =* 10^−7^ m^3^.sec^−1^. After the fractured network was fully saturated with distilled water, the three-way valve was rotated to enable the injection of distilled water seeded with tracer inside the fracture network. The distilled water seeded with tracer was injected for 15 seconds, keeping the flow rate constant to *Q =*10^−7^ m^3^.sec^−1^. After 15 seconds, the three-way valve was rotated again to enable distilled water injection. After two minutes of injection, which was the time it took for the tracer to reach the 3D-printed fracture network sample, we began to sample the fluid at the outlet every 10 seconds. The fluid samples were then analysed with a Sodium ion (Na^+^) concentration sensor (LAQUAtwin Na-11 (Horiba Co.)). We used the time series of the Sodium ion (Na^+^) concentration as tracer responses.

## Ethics Statements

The authors dully adhered to ELSEVIER ‘Ethics in publishing’ policy. No ethical issues are associated with this work.

## CRediT Author Statement

**Megumi Konno**: Investigation, Visualization, Writing - review & editing, **Alexandros Patsoukis Dimou:** Validation, Visualization, Writing - original draft, Writing review & editing. **Anna Suzuki**: Conceptualization, Writing - review & editing, Supervision.

## Declaration of Competing Interest

The authors declare that they have no known competing financial interests or personal relationships that could have appeared to influence the work reported in this paper.

## Data Availability

Measurement of porosity, permeability, and tracer response using 3D-printed fracture networks (Original data) (OSF (Open Science Framework)).

## References

[1] A. Patsoukis Dimou, H.P. Menke, J. Maes, Benchmarking the Viability of 3D Printed Micromodels for Single Phase Flow Using Particle Image Velocimetry and Direct Numerical Simulations, Transport in Porous Media (2021). https://doi.org/10.1007/s11242-021-01718-8.

[2] A. Patoukis Dimou, M. Konno, A. Suzuki, Measurement of porosity, permeability, and tracer response using 3D-Printed Fracture Networks OSF, 2023. 10.1016/j.dib.2023.109010 PMC9993027 36909018

[3] Suzuki A., Watanabe N., Li K., Horne R.N. (2017). Fracture network created by 3-D printer and its validation using CT images. Water Resour. Res..

[4] Vermilye J.M., Scholz C.H. (1995). Relation between vein length and aperture. J. Struct. Geol..

[5] Suzuki A., Niibori Y., Fomin S.A., Chugunov V.A., Hashida T. (2015). Fractional derivative-based tracer analysis method for the characterization of mass transport in fractured geothermal reservoirs. Geothermics.

[6] Watanabe K., Takahashi H. (1995). Fractal geometry characterization of geothermal reservoir fracture networks. Journal of Geophysical Research: Solid Earth.

[7] Suzuki A., Bjarkason E.K., Yamaguchi A., Hawkins A.J., Hashida T. (2022). Estimation of flow-channel structures with uncertainty quantification: Validation by 3D-printed fractures and field application. Geothermics.

[8] J. Bear, Dynamics of fluids in porous media, Courier Corporation 1988.

